# Bmp Indicator Mice Reveal Dynamic Regulation of Transcriptional Response

**DOI:** 10.1371/journal.pone.0042566

**Published:** 2012-09-11

**Authors:** Anna L. Javier, Linda T. Doan, Mui Luong, N. Soledad Reyes de Mochel, Aixu Sun, Edwin S. Monuki, Ken W. Y. Cho

**Affiliations:** 1 Department of Developmental and Cell Biology, School of Biological Sciences, University of California Irvine, Irvine, California, United States of America; 2 Department of Pathology and Laboratory Medicine, School of Medicine, University of California Irvine, Irvine, California, United States of America; Ecole Normale Supérieure de Lyon, France

## Abstract

Cellular responses to Bmp ligands are regulated at multiple levels, both extracellularly and intracellularly. Therefore, the presence of these growth factors is not an accurate indicator of Bmp signaling activity. While a common approach to detect Bmp signaling activity is to determine the presence of phosphorylated forms of Smad1, 5 and 8 by immunostaining, this approach is time consuming and not quantitative. In order to provide a simpler readout system to examine the presence of Bmp signaling in developing animals, we developed BRE-gal mouse embryonic stem cells and a transgenic mouse line that specifically respond to Bmp ligand stimulation. Our reporter identifies specific transcriptional responses that are mediated by Smad1 and Smad4 with the Schnurri transcription factor complex binding to a conserved Bmp-Responsive Element (BRE), originally identified among *Drosophila, Xenopus* and human Bmp targets. Our BRE-gal mES cells specifically respond to Bmp ligands at concentrations as low as 5 ng/ml; and BRE-gal reporter mice, derived from the BRE-gal mES cells, show dynamic activity in many cellular sites, including extraembryonic structures and mammary glands, thereby making this a useful scientific tool.

## Introduction

Bmp ligands are secreted growth factors that trigger activation of a highly conserved signaling circuit that is utilized throughout development, from the subdivision of tissue types during early embryogenesis to the formation of limbs and internal organs. Regulation of Bmp signaling activity is very dynamic and complicated, involving multiple layers of regulation both at the extracellular and intracellular levels. Extracellular modulators such as Chordin and Noggin are often expressed by the same or nearby cells to antagonize the Bmp signal [1], [2], [3], [4]. Intracellularly, transcriptional responses toward Bmp signaling can be further modulated by the presence of inhibitory Smad6 and 7, which antagonize the normal function of Smad1, 5 and 8, (Bmp R-Smads), or by altering the availability of these signal transducers within the cell [5], [6], [7], [8], [9]. Thus, the presence of Bmp ligands or Bmp signaling components is not an unequivocal indicator of Bmp activity. A common approach used to detect the spatial localization of Bmp activity is to perform immunostaining on embryos or tissues with antibodies that specifically recognize the phosphorylated forms of Smad1, 5 and 8 (P-Smad1/5/8). However, this approach can be tedious and time consuming, and has the drawback of not sensing the transcriptional response of a cell. Therefore, an additional tool to measure the transcriptional response of cells toward Bmp ligands would be beneficial in the Bmp biology field. We developed a simple readout system to examine the presence of Bmp signaling in both mouse embryonic stem (mES) cells, and a transgenic mouse line, that detects the transcriptional output mediated by a Bmp response element (BRE) we characterized previously [10], [11].

Bmp ligands binding to their receptors result in activation of the Bmp R-Smads in the cytoplasm. However, how Bmp R-Smads specifically recognize “target” genes for regulation remains poorly understood [12]. Previous studies showed that Smads 1 and 5 recognize short, specific GC-rich DNA sequence elements (GCCG-like motifs) [12], [13], [14], [15], [16]; and Smad4 binds the highly conserved SBE (Smad binding element, 5′-GTCT-3′) [17], [18]. Studies in *Drosophila* and *Xenopus* identified a BRE as a regulatory sequence found in various genes, including known Bmp targets such as the *Xenopus id3* and *ventx2* genes, and the *Drosophila brk* gene [10], [11]. The zinc finger Schnurri (Shn) protein can act as a co-factor with Bmp R-Smads to bind the BRE in a certain conformation, and elicit a transcriptional response [11], [19]. The proposed regulatory mechanism involves Smad1 and Smad4 complexing with Shn (Smad1/4-Shn) at the BRE, which requires a five nucleotide (nt) spacer separating the Smad binding sites, suggesting that the binding conformation of these factors is important for BRE-mediated modulation [11]. Our Bmp indicator mice use the BRE from the *Xenopus id3* regulatory sequence, which consists of Smad1 and Smad4 binding sites (5′-GACGCC-3′ and 5′-GTCTG-3′) separated by a five nt spacer. A *lacZ* reporter gene was used to reveal areas of BRE-mediated activity of BMP ligands, and this transgene is hereafter referred to as BRE-gal.

In this study, we use our BRE-gal indicator mice to characterize the subset of Bmp activity that is modulated by this regulatory element. Since this motif is found in various genes of many organisms, we hypothesized that the BRE-gal reporter will respond to a subset of Bmp responding cells where BRE-mediated transcriptional response is functional. Our expression analysis of the BRE-gal mouse reveals that many sites of Bmp activity utilize the BRE to control the specific and dynamic roles of this growth factor during development, thereby making this a useful scientific tool. Interestingly, we also find robust expression of the BRE-gal reporter in extraembryonic structures and mammary glands, suggesting that during vertebrate evolution BRE-mediated Bmp responses were co-opted to regulate development of these structures.

## Results and Discussion

### Generation and response of mouse ES cells harboring BRE reporter genes

**Figure 1:**
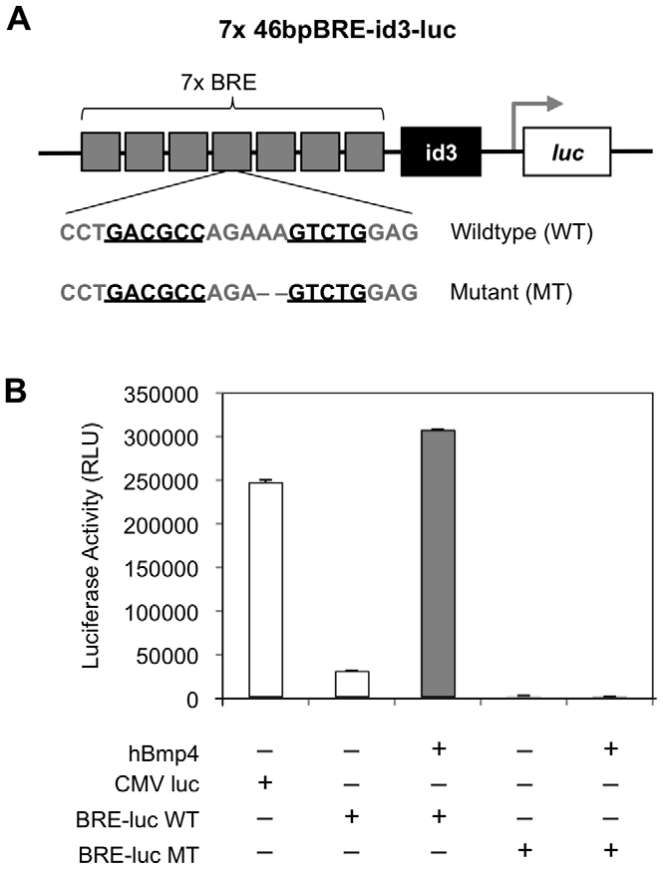
BRE-mediated responsiveness depends on the five nucleotide spacer between Smad binding sites. (A) Luciferase reporter constructs were generated with either the wildtype BRE sequence (BRE-luc WT) containing a five nucleotide (nt) spacer, or a mutant BRE sequence (BRE-luc MT) containing a two nt deletion in the spacer. In the diagram, the Smad binding sites are underlined. (B) Transient luciferase assays showed that Bmp responsiveness in mES cells was abrogated if the length of the spacer is decreased. BRE-luc WT and BRE-luc MT constructs were transfected into wildtype mES cells. Twenty-four hours after transfection, the cells were treated with or without Bmp4 at 10 ng/ml for six hours. BRE-mediated responsiveness in BRE-luc WT mES cells increased after treatment with Bmp4.

Previously we demonstrated that the frog BRE functions in *Drosophila*, *Xenopus* and zebrafish [10], [11], [20]. We therefore tested whether the same *Xenopus* BRE responds to Bmp signaling in mES cells. The BRE(7X)-luc construct harbors seven copies of the BRE driving a −201/+70 *Xenopus id3* minimal promoter and a *luciferase* reporter gene. To confirm whether Bmp responsiveness is dependent on the binding conformation of the Smad1/Smad4 and Shn complex at the BRE, we compared luciferase activation from a wild type (WT) and mutant (MT) BRE sequence (Figure 1A). Deletion of two nucleotides in the 5 nt spacer between Smad1 and Smad4 binding sites of the BRE provided the sequence used in the mutant BRE-luc reporter, which also contains a multimerized version of the mutant BRE sequence. Previously, it was shown that decreasing the spacer length interfered with the binding of Smad1/Smad4 and Shn to the BRE DNA sequence in both *Drosophila* and *Xenopus* embryos [11], [13], [19]. Therefore, we examined whether the same BRE motif via a Smad1/Smad4/Shn interaction was extended to mammalian systems. Each reporter construct was transfected into feeder-independent E14 mES cells, then stimulated with Bmp4 for four hours. When luciferase activity was measured, the mutant BRE reporter failed to respond to Bmp4 while the wild type BRE-luc responded well (Figure 1B). These results suggest that mES cell response to Bmp signaling requires proper spacing of Smad1 and 4 binding sites within the BRE, strengthening the evolutionarily conserved function of BRE-mediated Bmp signaling between *Drosophila*, *Xenopus*, zebrafish and mammals [10], [11], [20].

**Figure 2:**
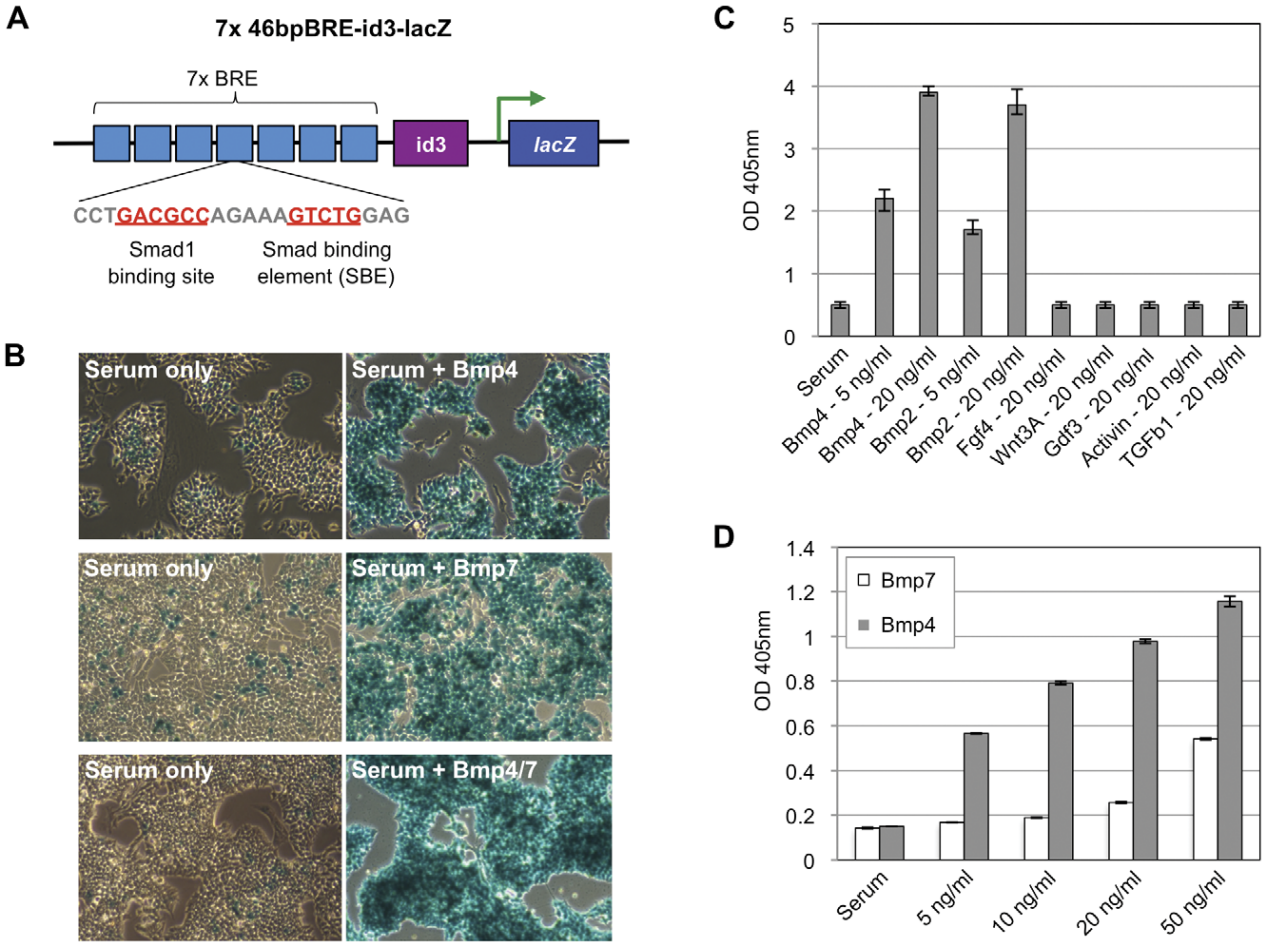
The BRE-gal reporter mES cell line can respond to various Bmp ligands. (A) The *Xid3* BRE consists of a Smad1 binding site (5′-GACGCC-3′) and a highly-conserved Smad Binding Element (SBE, 5′-GTCTG-3′) for Smad4 binding, separated by a 5-nucleotide spacer. In the diagram, the Smad binding sites are indicated in red and underlined. (B) BRE-gal mES cells were treated with the indicated Bmp ligands, and then stained with X-gal. Column 1 shows reporter response without addition of exogenous Bmp ligand to the culture media. Column 2 shows reporter response after addition of Bmp ligand. Bmp4 and Bmp4/7 were added at 10 ng/ml, and Bmp7 was added at 50 ng/ml. Magnification is at 20×. It should be noted that (C) BRE-gal mES cells were treated with the indicated growth factors and concentrations. There is an increased, dose-dependent response to Bmp2 and Bmp4, compared to other growth factors. (D) BRE-gal mES cells respond more strongly to Bmp4 than Bmp7 at each indicated concentration. Reporter cells were treated with recombinant hBmp4 or hBmp7 for 24 hours at the indicated concentrations. Quantification of *lacZ* expression was quantified using an enzymatic assay with the colorimetric lactose analog ONPG.

Next, we generated a stable mES cell line harboring a *lacZ* (ß-galactosidase) reporter gene driven by a multimerized *Xenopus id3* BRE (Figure 2A). A Wnt-responsive nuclear ß-galactosidase (BAT-gal) reporter gene [21] was modified by replacing the minimal promoter region and Tcf/Lef binding sites with a minimal *Xenopus id3* promoter containing the multimerized BRE. The *lacZ* open reading frame (ORF) used in the reporter encodes a nuclear localization signal so that individual cells exhibiting BRE-gal reporter activity can be identified by their nuclear staining. E14 mES cells were electroporated with the BRE-gal reporter, and selected for neomycin resistance. Single-copy integration of the transgene was confirmed by Southern blot analysis. Following karyotype analysis to verify normal chromosome count, two BRE-gal mES cell lines were further analyzed.

Stimulation of these reporter cells with homodimers of Bmp2, 4, and 7, and heterodimer Bmp4/7 elicited a uniform transcriptional response (Figure 2B–C). However, we note that some cells in the serum-only condition stained for X-gal, perhaps due to autocrine secretion of Bmp ligands from neighboring mES cells. The BRE-gal response was detected at concentrations as low as 2 ng/ml of Bmp2, 4 and Bmp4/7, but using concentrations higher than 5 ng/ml gave more reliable, homogeneous, and robust BRE-gal reporter expression (Figure 2B, data not shown). In BRE-gal mES cells, 10 ng/ml of Bmp4/7 heterodimer was able to yield a homogenous BRE-gal response, but a higher dose of 50 ng/ml of Bmp7 homodimer was used to obtain a similar result (Figure 2B). Comparison of homodimers Bmp4 and 7 also showed that Bmp4 elicited a stronger transcriptional response than Bmp7 at various doses of each ligand (Figure 2D). Non-Bmp growth factors, Fgf4 and Wnt3A, failed to activate the *lacZ* reporter (Figure 2C). The reporter mES cells also failed to respond to non-Bmp members of the Tgfß family such as Tgfß1, activin and Gdf3 (Figure 2C), suggesting that the BRE-gal response is specific to Bmp signaling. Since our BRE-gal mES cells cultured in serum does not respond to the low level of Bmp signaling that is normally present in serum [22] (see also Figure 2B), we suggest that BRE-gal mES cells respond to a range of intermediate to high concentrations of Bmp ligands.

### Dynamic BRE-gal expression during mouse embryogenesis

**Figure 3:**
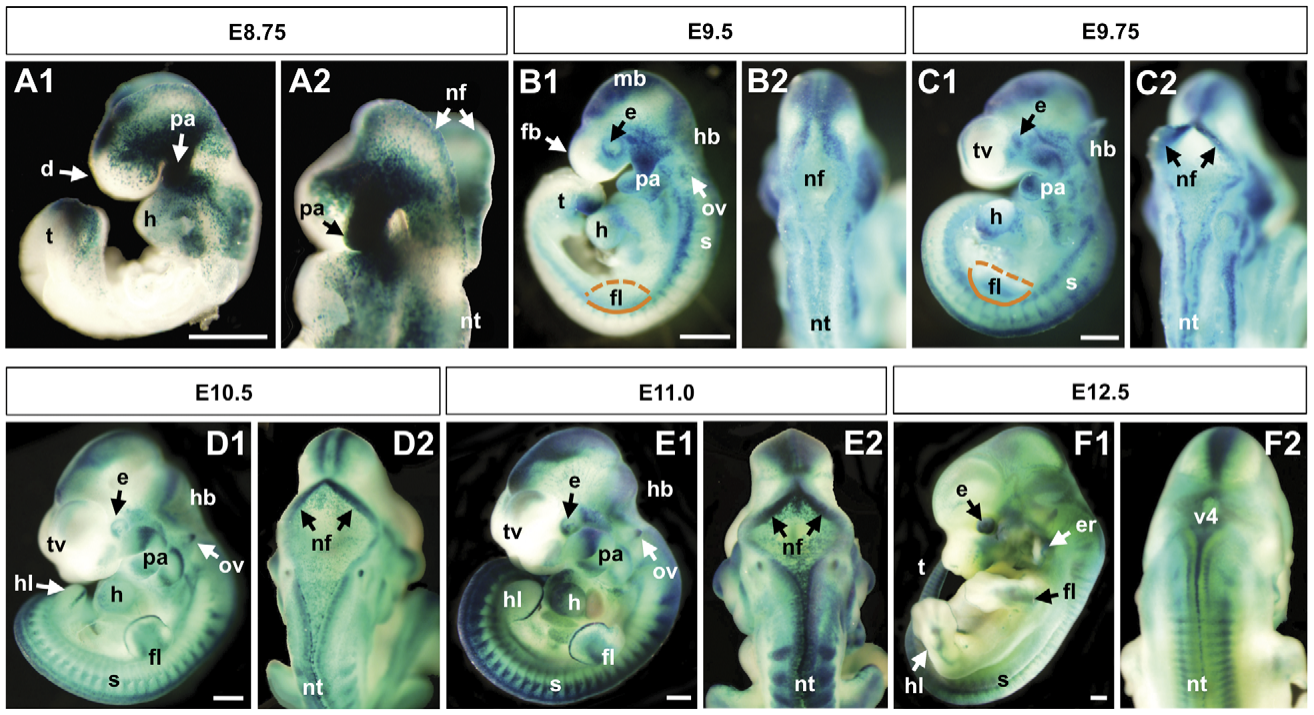
BRE-gal reporter activity during mid-gestation stage BRE-gal mouse embryos (E8.75–12.5). Wholemount X-gal staining of BRE-gal mouse embryos showed dynamic BRE-dependent Bmp signaling in various regions and tissues throughout development. Embryos are shown in left, lateral views (A1, B1, C1, D1, E1, F1). At E8.75, a dorsal-oblique view of open neural folds (nf) at the site of the future midbrain and hindbrain is shown (A2). Dorsal views of the neural tube are shown (B2, C2, D2, E2, F2). Abbreviations: d, diencephalon; e, eye; er, ear; fb, forebrain; fg, foregut; fl, forelimb bud; h, heart; hb, hindbrain; hl, hindlimb bud; mb, midbrain; nf, neural folds; nt, neural tube; ov, otic vesicle; pa, pharyngeal arch(es); s, somites; t, tailbud; tv, telencephalic vesicles; v4, fourth ventricle. Scalebar 0.5 µm.

In order to study BRE-mediated Bmp activity during mouse embryogenesis, BRE-gal mES cells were injected into mouse blastocysts, and these were implanted into foster mothers. BRE-gal mouse lines were established as described in the Materials and Methods. BRE-gal expression was consistently detected in a number of tissues, including heart, neural tube, and AER of the limb (Figure 3). However, non-transgenic embryos completely lacked X-gal staining (data not shown). Expression of our BRE reporter in pharyngeal arches, brain, heart, and eyes was similar to BRE-gfp transgenic reporter expression in *Xenopus laevis* and zebrafish embryos (Figure S1), suggesting that the pattern of BRE-mediated Bmp signaling response is an evolutionarily conserved mechanism.

For the analysis presented here, we first highlight certain organs and structures during mid-gestation stage embryogenesis (E8.75–E13.5) and discuss the BRE-gal expression patterns. Temporal staging of BRE-gal embryos at E8.75–E13.5 is in accordance with Kaufman (1992) [23]. Analysis of BRE-gal expression patterns during mid-gestation development is followed by a discussion of reporter expression during earlier pre-gastrulation through headfold stages (E5.5–E8.0).

### BRE-gal expression patterns in mid-gestation stage (E8.75–E13.5) embryos

#### A) Neural structures

**Figure 4:**
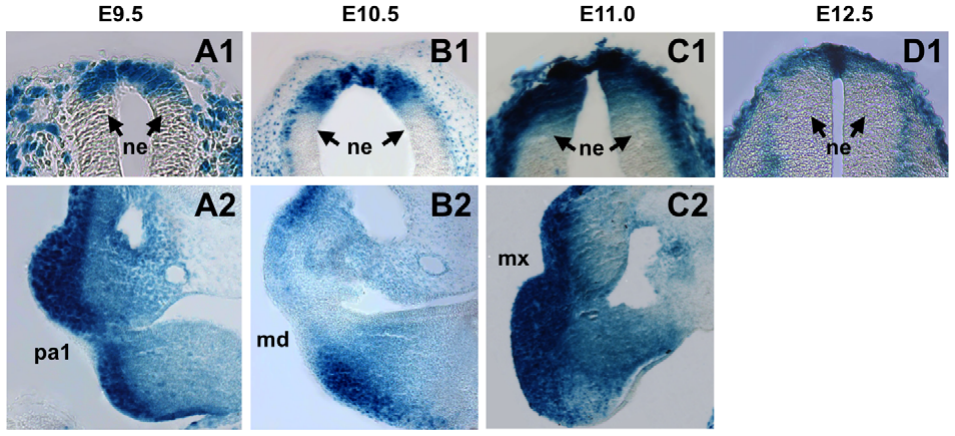
Sections showing BRE-gal reporter activity in various regions of mid-stage BRE-gal mouse embryos. Transverse sections (10–12 µm thickness) of wholemount X-gal stained BRE-gal embryos (E9.5–E12.5) are shown. The dorsal neural tube in the rostral region is shown, with dorsal facing up (A1, B1, C1, D1). The right pharyngeal arches are shown (A2, B2, C2). Abbreviations: md, mandibular component of first branchial arch; mx, maxillary component of first branchial arch; ne, neural ectoderm; pa1, first pharyngeal arch.

The vertebrate central nervous system develops from the neural plate, and initial induction of neural ectoderm requires inhibition of Bmp signaling. Morphogenesis of the neural plate into a tube begins at approximately E8.5, and appears to be governed predominantly by a balance between Bmp signaling and Bmp antagonism mediated by Noggin in the future dorsal region of the neural tube [24]. While *Noggin* expression has been observed in the tips of the neural folds and along the dorsal midline of the neural tube during closure [24], [25], our reporter showed BRE-gal activity in this region at E8.75 (Figure 3, panel A2; data not shown). This suggests that sufficiently high concentrations of Bmp growth factors are available in the dorsal midline despite the presence of Noggin. Alternatively, BRE-gal expression in this region at E8.75 may represent perdurance of the ß-galactosidase activity from earlier stages. BRE-gal activity continued in the dorsal midline of the neural tube during E9.5–E12.5 (Figure 3, panels B2, C2, D2, E2, F2), which was further examined in transverse sections (Figure 4, panels A1, B1, C1, D1). At E9.5 and E10.5, BRE-gal expression was confined to the dorsal cells of the neural tube, and the expression boundaries appeared to be sharp (Figure 4, panels A1, B1). At E11.0, strong BRE-gal activity extended ventrally along the lateral (outer) edge of the neural tube, and less intense BRE-gal activity was detected in the medial (inner) portion (Figure 4, panel C1). At E12.5, overall BRE-gal expression was robust in the dorsal midline cells, and has decreased in the rest of the dorsal neural tube (Figure 4, panel D1).

In agreement with our BRE-gal reporter data, expression of *Bmp4*, *5*, and *7* was detected in dorsal midline cells, and *Bmp7* expression was also observed in the overlying ectoderm [26]. Studies also showed that dorsal-ventral patterning of the neural tube occurs by morphogen gradients of Bmp and Wnt from the dorsal region, and Sonic hedgehog (Shh) from the ventral region [27], [28]. The intense BRE-gal staining in the midline was, therefore, consistent with the model that proposed a positive feedback circuit between Bmp and Wnt signaling pathways in the dorsal midline [29], [30]. In summary, between E8.75–E12.5 our reporter reveals a dynamic change in BRE-mediated Bmp activity in the developing neural tube.

The anterior-most region of the neural tube is patterned into the forebrain, midbrain, and hindbrain. After neural tube closure, the telencephalon arises from the forebrain and subdivides into left and right hemispheres. Coexpression of *Bmp2*, *4*, *5*, *6*, and *7* has been observed in the dorsal midline before separation of the hemispheres [31], while the receptors *Bmpr1a* and *Bmpr1b* were expressed more broadly [32]. During E9.5–E12.5, BRE-gal reporter activity was observed in the dorsal telencephalon midline (Figure 3), and a detailed analysis of this expression pattern is provided in our companion publication, Doan et al. (submitted).

#### B) Pharyngeal arches

**Figure 5:**
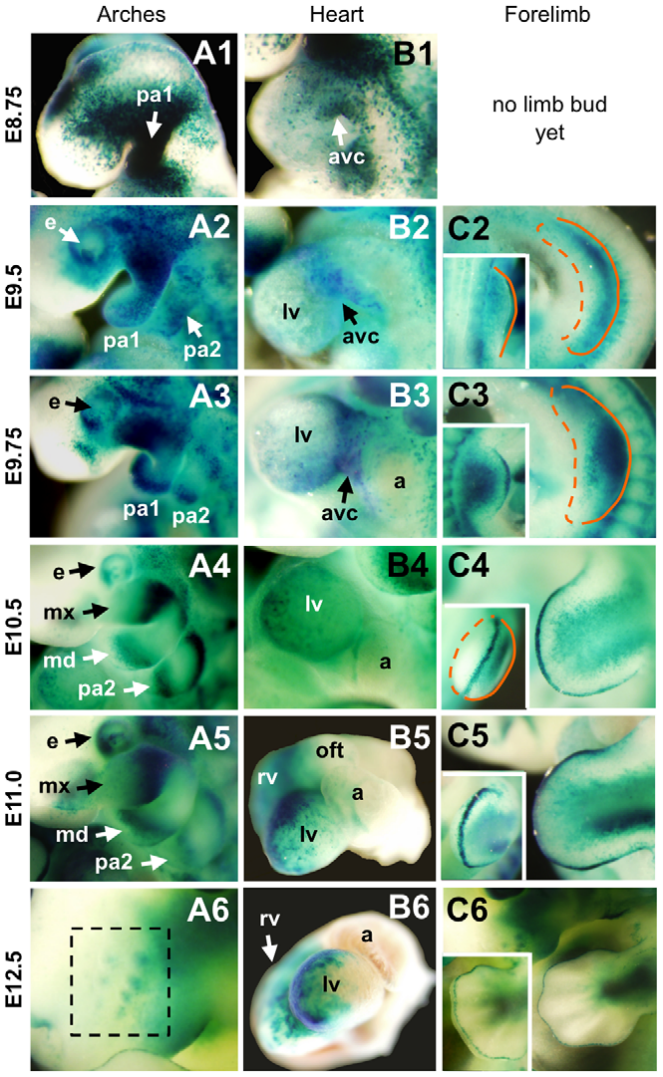
BRE-gal reporter activity during mid-gestation stage BRE-gal mouse embryos (E8.75–12.5). Magnified view of various structures in wholemount X-gal stained BRE-gal embryos are shown. Only the first pharyngeal arch is present at E8.75 (A1), and the second pharyngeal arch follows (A2–A5). By E10.5, the maxillary and mandibular components of the first branchial arch are apparent (A4–A5). At E12.5, the vibrissal follicle placodes (shown within the black, dashed box) appear on the snout, which develops from the maxillary component of the first branchial arch (A6). While the heart tube (B1) forms and loops, the atria and ventricles start to develop as well (B2–B5). At E9.5, the forelimb buds can be seen protruding laterally from the trunk and continue to grow outward (C2–C3, dorsal faces right, indicated with a solid orange line; ventral faces left, indicated with a dashed orange line). BRE-dependent Bmp activity appears primarily on the dorsal side (inset C2–C3, distal edge outlined with solid orange line). By E10.5, the forelimb buds are more prominent (C4–C5, dorsal view). By E12.5, the future digits of the handplate are visible (C6, dorsal view). The apical ectodermal ridge (inset, C4–C6) runs along the dorsal-ventral boundary of the forelimb. Abbreviations: a, atrium; avc, atrioventricular canal; e, eye; lv, left ventricle; nf, neural folds; mx, maxillary component of first branchial arch; nt, neural tube; pa1, first pharyngeal arch; pa2, second pharyngeal arch.

Neural crest (NC) cells from the dorsal neural tube comprise the majority of cells in the pharyngeal arches, and migration of these NC cells into the first arch is complete by E9.0. In accordance with this notion, our BRE-gal mice revealed a robust and dynamic pattern of reporter activity in the pharyngeal arches during E8.75–E11.0 (Figure 4, panels A2-C2; Figure 5, panels A1–A5).

At E9.0, the expression of *Tfapa*, a NC cell marker, highlights the NC cells migrating into the first pharyngeal arch [33]. At a similar stage, our BRE-gal reporter showed intense, uniform staining in the first pharyngeal arch (pa1) that was continuous with the adjacent cephalic domain (Figure 5, panel A1). Interestingly, this expression of our BRE-gal reporter was strikingly similar to the expression of *Tfap2a* in migratory NC cells. This suggests that BRE-regulated Bmp signaling may also be involved in NC cell migration and/or specification into the first pharyngeal arch.

As development progressed, the strong and uniform *lacZ* reporter expression in the pharyngeal arches gave way to a more restricted pattern (E9.5, Figure 5, panel A2), and at E9.75, intense reporter activity was restricted to the distal and proximal regions of each arch (Figure 5, panel A3). At E10.5–E11.0, this distal and proximal BRE-gal expression pattern was present in the mandibular (pa1, md, Figure 4, panel B2; Figure 5, panel A4) and hyoid arches (pa2, Figure 5, panels A4, A5). In the maxillary arch, BRE-gal activity was confined to the proximal portion (mx, Figure 5, panels A4, A5).

Our BRE-based data in murine pharyngeal arches is comparable to previous BRE studies in zebrafish and Xenopus pharyngeal arches. A similar pattern of BRE activity in the pharyngeal arches was demonstrated in transgenic zebrafish using a five concatamer sequence of the BRE isolated from the *Xenopus ventx2* gene [10], [20]. In these BRE-gfp zebrafish, GFP was observed throughout the embryonic arches, with strongest expression in the ventral portion (corresponding to distal region in the mouse). Our previous study of transgenic *Xenopus* embryos using a multimerized BRE driving a GFP reporter gene also revealed expression in the pharyngeal arches [10]. Our mouse BRE-gal reporter corroborated that a higher level of Bmp signaling occured in the distal domain. However, during mouse stages E10.5–E11.0 our reporter also showed strong BRE-gal expression in the proximal region during pharyngeal arch development (Figure 5, panels A4, A5). This may be related to development of the peripheral nervous system, as Bmp ligands were required for induction of epibranchial ganglia, which was demonstrated in zebrafish [34]. Because a BRE-dependent mechanism of Bmp signal regulation is utilized in the pharyngeal arch develop of mice, zebrafish, and *Xenopus*, this strengthens the notion that this regulatory mechanism is evolutionarily conserved.

#### C) Heart

It is well established that Bmp signaling is important at multiple stages of cardiogenesis. Our BRE-gal reporter showed varying activity throughout the developing heart starting at E8.75, Figure 5, panels B1–B6). However, the exact spatial and temporal locations of Bmp signaling are still unclear. This is further complicated by the functional redundancies among Bmp signal components that obscure analyses of Bmp ligands in this process.

Formation of the cardiac crescent at approximately E7.0 in mice depends on signaling through the type I receptor, Bmpr1a [35]. Bmp2 transcripts were observed here [36], and expression of Bmp targets, *Nkx2.5* and *Gata4*, were reported in the cardiac crescent [37]. Despite evidence for the presence of Bmp signaling activity in the cardiac crescent, our BRE-gal reporter showed almost no staining in this structure (see a more detailed discussion of the cardiac crescent in the subsequent section “Early and late headfold stages (E7.5–E8.0)”). Later, at E8.75 our reporter showed BRE-gal activity throughout the heart tube, with strong activity in the developing atrioventricular canal (avc), which continued until E9.75 (Figure 5, panels B1–B3). Consistent with this expression pattern, a high level of Bmp2 transcript was detected in the avc at E9.0 [38]. Thus, while Bmp2 transcripts were seen in the cardiac crescent and avc, BRE-mediated activity was observed only in the avc. This suggest that differential levels of Bmp signaling activities exist in these two tissues, or alternatively a Shn- and BRE-independent mechanism is utilized in the cardiac crescent.

In vertebrates, the emergence of the avc marks the progression of the heart tube into a chambered organ. Low level BRE-gal activity was detected throughout the transitioning heart tube during E9.5–E9.75 (Figure 5, panels B2–B3), which was in agreement with low level *Bmp7* expression throughout the developing heart [38]. By E10.5, the heart chambers are evident, and BRE-gal expression was particularly high in the ventricles during E10.5–E12.5 (Figure 5, panels B4–B6), suggesting a regionalization of BRE-dependent Bmp activity. Also, at E10.5–E12.5, BRE-gal reporter activity was observed in the proximal region of the outflow tract (oft, Figure 5, panel B5; data not shown). At E11.5, broad expression of *Bmp2*, *5*, *7*, and *Acvr2* was observed in the outflow tract [39]. Again, this suggests that the BRE can be utilized to mediate a subset of Bmp signaling within a tissue or organ.

#### D) Limbs

BRE-mediated Bmp activity was detected in the apical ectodermal ridge (AER) at E9.75–E12.5 (Figure 5, panels C3–C6). The AER is an ectodermal thickening at the distal edge of the limb bud that is crucial for proper limb outgrowth, and it distinguishes the boundary between the dorsal and ventral sides of the limb. The BRE-gal expression pattern in the AER was in agreement with independent data that identified Bmp2, 4, 7 transcripts in this structure [40], [41], [42], and implicated Bmp signaling through Bmpr1a in limb ectoderm as important for establishing the AER [43]. In further support of our BRE-gal data, the AER-expressed *Id2* gene is a Bmp target that contains an almost identical BRE sequence (GACGCCNNNNNGTCTG for *Id3* vs. GGCGCCNNNNNGTCTG for *Id2*) in the regulatory regions of the human and mouse homologs [44], [45]. In addition, the mouse *Flrt3* gene has two BRE-like sequences, and its expression in the AER is important for maintaining this structure during proper limb outgrowth [46]. Thus, the BRE module may be regulating a cohort of Bmp target genes in the AER.

At E9.5, we found that BRE-gal reporter activity was stronger in the dorsal forelimb (Figure 5, panel C2), whereas other studies showed *Bmp2* expression in the posterior mesenchyme and ventral ectoderm [41], *Bmp4* expression throughout the mesoderm and ventral ectoderm [42], and *Bmp7* expression throughout the mesenchyme and ectoderm [40]. The different expression patterns for each ligand suggests varying roles for Bmp growth factors throughout the limb bud at E9.5, and underscores the importance of differential transcriptional responses (BRE-dependent and -independent) in different BMP signaling regions. At E10.5, our BRE-gal reporter showed staining in the limb bud mesenchyme that was bordered by darker anterior and posterior stripes (Figure 5, panel C4); and later at E11.0, the posterior stripe became more intense than the anterior stripe (Figure 5, panel C5, Figure S2). This expression pattern hints at a role for BRE-mediated Bmp activity in anterior-posterior patterning of the limb. Bmp4 transcripts were observed only in the mesoderm immediately underlying the AER at E10.5–E12.0 [42]. This suggests that other Bmp ligands may be involved in anterior-posterior patterning of similar stage limb buds (E10.5–E11.0, Figure 5, panels C4–C6, Figure S2).

It has been established that Bmp activity is responsible for apoptosis in the interdigital mesenchyme to refine the digits [41], [47]. The Bmp-responsive mouse *Id2* gene is an apoptosis-promoting candidate that is expressed in this tissue during digit formation [44], [48]. However, our reporter did not show any activity in the interdigital mesenchyme at E12.5 (Figure 5, panel C6). Despite the presence of an almost identical BRE in the *Id2* regulatory region, this raises the possibility that transcriptional activation of *Id2* within interdigital mesenchyme is regulated by a non-BRE-dependent mechanism. This highlights the intricate way a gene can be regulated in different contexts, and supports the notion that the BRE modulates only a subset of Bmp signaling.

Lastly, our BRE-gal reporter showed robust activity along the limb, excluding the digits (Figure 5, panel C6). There is ample evidence demonstrating the role of Bmp signaling in limb skeletogenesis [49], [50], [51], [52], [53], [54], and our reporter suggests that skeleton formation in the limb, but not the digits can be modulated by the BRE. Although the hindlimb develops slightly later, the BRE-gal expression pattern is identical to the forelimb (Figure 3, panels D1, E1, F1; data not shown). In summary, our BRE-gal indicator has uncovered a complex Bmp-mediated transcriptional response underlying limb bud development.

#### E) Ectodermal appendages

**Figure 6:**
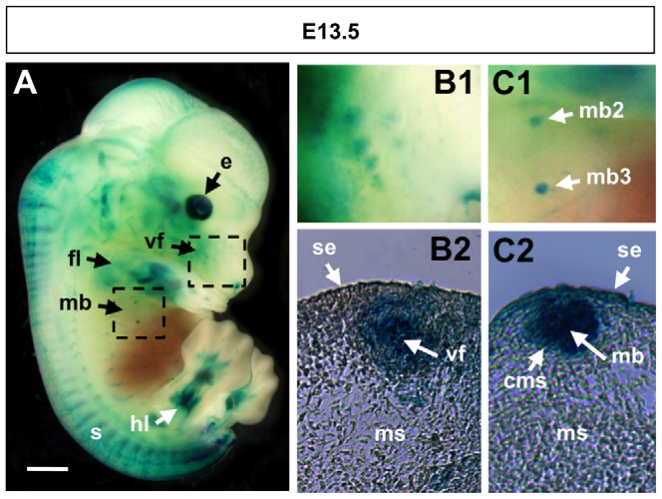
BRE-gal reporter activity in the mammary buds and vibrissal follicles of an E13.5 mouse embryo. A wholemount X-gal stained BRE-gal mouse embryo at E13.5 is shown in a right, lateral view (A). The upper, right dashed box in (A) indicates the area that is magnified in (B1), and a transverse section of a vibrissal follicle is shown in (B2). The lower, left dashed box in (A) indicates the area that is magnified in (C1), and a transverse section of a mammary bud is shown in (C2). Sections are 12 µm thickness. Abbreviations: cms, condensing mesenchyme; fl, forelimb bud; hl, hindlimb bud; mb, mammary buds; mb2, second mammary bud on right side; mb3, third mammary bud on right side; ms, mesenchyme; s, somites; se, surface ectoderm; vf, vibrissal follicles. Scalebar 1 mm.

Ectodermal appendages include such structures as the mammary glands and hair follicles. While these two tissues are vastly different, their initial morphological development is similar: local epithelial thickenings form a placode that will then invaginate into a bud, around which the adjacent mesenchyme condenses [55]. Constant signaling between the surface ectoderm and mesenchyme is governed by various signals, including the Wnt, Fgf, and Tgfß families. At E12.5–13.5, our BRE-gal reporter showed activity in the vibrissal follicle placodes and mammary buds (Figure 6).

The vibrissae (whiskers) are a specialized hair type optimized for sensing, although their differentiation is essentially the same as for pelage hair. The maxillary component of the first branchial arch gives rise to the snout, and development of the vibrissal follicles initiates on the snout at E12.5. At E12.5–13.5, we observed BRE-gal activity in the vibrissal follicle placodes, but not in the adjacent surface ectoderm (Figure 5, panel A6; Figure 6, panels B1, B2). In support of our findings, Bmp2a transcripts were observed in the whisker placodes of E13.5 mice [41]. In addition to Bmp signaling, Fgf signaling has been implicated in vibrissal follicle placode formation as evidenced by Fgf10 mutant mice with a reduced number of follicles, and an aberrant follicle polarity when the whiskers formed [56].

The majority of mammary gland development occurs during adulthood; but rudimentary glands form during embryogenesis, and further development is arrested until puberty. In the female mouse, embryonic development of the five, paired mammary glands occurs between E10.5–E18.0 [57]. From the surface ectoderm-derived mammary lines, the placodes form in a non-sequential manner: pair 3 develops first, followed by pair 4, then pairs 1 and 5 appear simultaneously, and pair 2 forms last [58], [59]. The placodes then invaginate into buds that are surrounded by condensing mesenchyme. At E12.5–E13.5 our BRE-gal reporter showed distinct staining in three epithelial mammary buds (corresponding to pairs 2, 3, 4), and in the overlying ectoderm (Figure 6, panels C1, C2). For unstained mammary pairs 1 and 5, the overlying ectoderm was also free of reporter gene expression (data not shown). This hints at an interesting mechanism in which the embryonic mammary glands are not all regulated in the same manner. In support of this notion, mice defective for *Fgf10* failed to form mammary placode pairs 1, 2, 3, 5, whereas pair 4 was unaffected [59]. Furthermore, in mice mutant for the receptor *Fgfr2b*, mammary placode pair 4 initially formed, but was not maintained beyond E12.5 [59].

In summary, the roles of Bmp signaling in vibrissal and mammary placodes are yet to be confirmed. However, based on the expression patterns of our BRE-gal reporter, we hypothesize that Bmp signaling, possibly interacting together with Fgf signaling, contributes to the development of ectodermal appendages.

### Dynamic BRE-gal expression during pre-gastrulation through headfold stages of early mouse embryogenesis

During gastrulation, the rate of embryonic development can vary between litters and even within the same litter. As a result, temporal staging of these embryos can be inaccurate. Therefore, we also use the morphological landmarks described in Downs and Davies (1993) [60], which allows for more accuracy and comparability.

### Pre-primitive streak and primitive streak stages (E5.5–E6.5)

**Figure 7:**
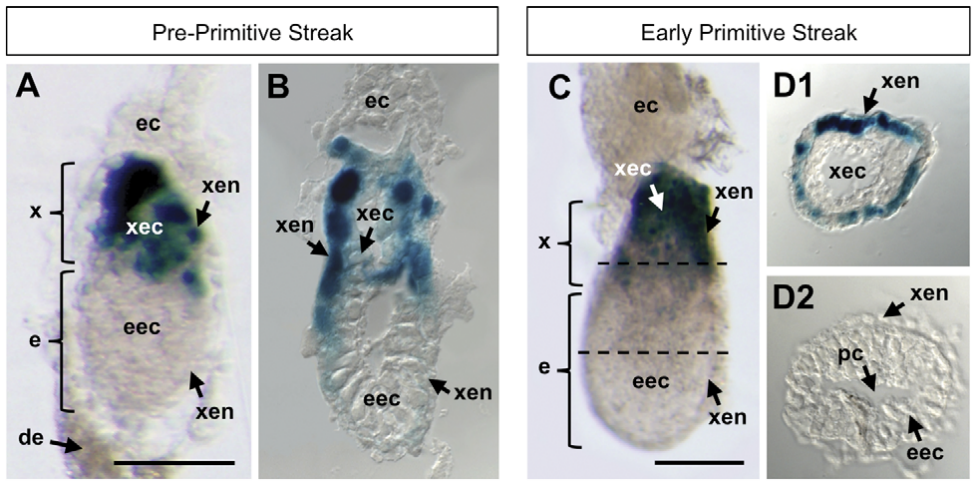
BRE-gal reporter activity in mouse embryos at pre-primitive streak and early primitive streak stages (E5.5–6.5). Wholemount X-gal staining of BRE-gal mouse embryos showed BRE-dependent Bmp signaling in extra-embryonic structures. A pre-primitive streak embryo (E5.5) is shown in (A), with a corresponding sagittal section (B). The plane of this section is slightly oblique. An early primitive streak embryo (E6.5) is shown in (C), with corresponding transverse sections through the (D1) extraembryonic and (D2) embryonic regions. The dashed lines in (C) indicate the plane of the sections shown in (D1, D2). All sections are slightly enlarged with respect to the corresponding image of the whole embryo. Sections are 10 µm thickness. Abbreviations: de, decidua; e, embryonic region; ec, ectoplacental cone; eec, embryonic ectoderm; pc, proamniotic cavity; x, extraembryonic region; xec, extraembryonic ectoderm; xen, extraembryonic visceral endoderm. Scalebar 200 µm.

At E5.5, the mouse embryo is elongating from the implanted blastocyst to the gastrulating egg cylinder. This early embryo consists of extraembryonic ectoderm (xec) adjacent to embryonic ectoderm (eec), and surrounded by extraembryonic visceral endoderm (xen). The proamniotic cavity initiates within the eec; and as this cavity elongates, the eec forms its characteristic cup shape. At E5.5, BRE-gal activity was observed in the xen overlying the xec (Figure 7A–B), which was in agreement with another study that detected P-Smad1/5/8 in the overlying xen [61].

At E6.5, the embryo is often described as an egg cylinder, and gastrulation is marked by the appearance of the primitive streak. During this early primitive streak stage, we continued to observe BRE-gal activity in the xen overlying the xec (Figure 7, panels C, D1, D2). This BRE-gal pattern was different from another report that showed the absence of P-Smad1/5/8 in the xen at E6.5 [61]. While we cannot resolve this difference at present, the detection of Bmp2 and Smad1 transcripts throughout the xec [62], [63], and the morphological defects in the xen of Smad1-deficient embryos [63] supports the notion that Bmp signaling is active in extraembryonic tissues.

It is well established that at approximately E5.5–E6.5, Bmp4 signaling from the xec to the proximal eec is important for inducing primordial germ cells (PGCs) [63]. Bmp4 transcripts were initially present throughout the xec, but at E6.5 Bmp4 transcripts became restricted to the region immediately abutting the eec [64]. In addition, *Bmp2* (expressed in the xen) and *Bmp8b* (expressed in the xec) cooperated with Bmp4 to generate PGCs [64], [65], [66]. The combined action of these Bmp signals takes place in the proximal eec, as evidenced by the presence of P-Smad1/5/8 in this region [61]. Because our BRE-gal reporter did not show expression in the eec at E5.5–E6.5, this suggests that the genes involved in PGC formation are not regulated by the BRE. Alternatively, the lack of BRE-gal activity in the eec could be due to the limited sensitivity of the reporter.

### Early and late headfold stages (E7.5–E8.0)

**Figure 8:**
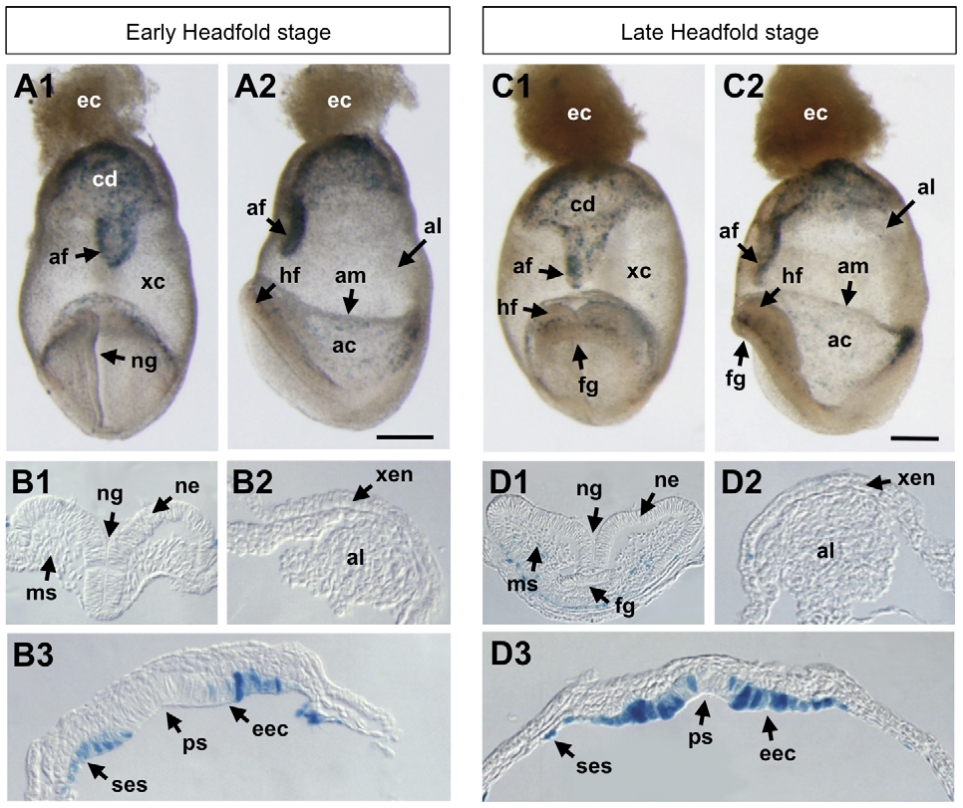
BRE-gal reporter activity in mouse embryos at headfold stages (E7.5–8.0). Wholemount X-gal staining of BRE-gal mouse embryos showed BRE-dependent Bmp signaling in embryonic and extra-embryonic structures. Anterior view of early headfold stage embryo (E7.5–E8.0) is shown in (A1), with a corresponding lateral view (A2), with anterior at left. Transverse sections of the early headfold stage embryo are shown in (B1, B2, B3). Anterior view of late headfold stage embryo (E7.5–E8.0) is shown in (C1), with a corresponding lateral view (C2), with anterior at left. Transverse sections of the late headfold stage embryo are shown in (D1, D2, D3). All sections are 10 µm thickness. Abbreviations: ac, amniotic cavity; af, amniotic fold; al, allantois; am, amnion; cd, chorionic dome; ec, ectoplacental cone; eec, embryonic ectoderm; fg, foregut diverticulum; hf, headfolds; ms, mesenchyme; ne, neural ectoderm; ng, neural groove; ps, site of primitive streak; ses, surface ectoderm and somatopleure; xc, exocoelomic cavity; xen, extraembryonic visceral endoderm. Scalebar 200 µm.

Our BRE-gal reporter mice continued to display *lacZ* reporter expression in extraembryonic tissues at late gastrulation (Figure 8, panels A1, A2, C1, C2). During early and late headfold stages (E7.5–E8.0), BRE-gal activity was detected in the chorionic dome, amniotic fold, and amnion. The chorion and amnion are distinct and separate extraembryonic membranes that enclose the developing embryo. At this stage, the chorion is comprised of xec and extraembryonic mesoderm; and the amnion is composed of primitive ectoderm and extraembryonic mesoderm. BRE-gal reporter activity in the amnion and chorion was in agreement with previous findings that showed *Bmp2* expression in these same tissues [67], [68]. Interestingly, Smad5-deficient embryos show disrupted amnion development, along with increased expression of *Bmp2* and *4* in the amnion [69]. These findings are consistent with the role of Bmp signaling in amnion development.

During gastrulation, extraembryonic mesoderm intercalates between the xec and primitive ectoderm to form the extraembryonic mesoderm-lined exocoelomic cavity (xc, Figure 8, panels A1, C1). Bmp2, and Smad1 and 5 transcripts were observed in the extraembryonic mesoderm of the xc [63], [68]. However, we detected no BRE-gal activity in this tissue layer. The allantois arises from extraembryonic mesoderm; and it elongates through the xc to fuse with the chorion and contribute to the placenta [70]. Previous findings demonstrated *Bmp2* and *4* expression in the allantois [64], [65], [68]. Additionally, in Bmp2 null mouse embryos, allantois development was delayed (in some cases the allantois did not fuse with the chorion), and the amnion and chorion did not form properly because the proamniotic canal failed to close [68]. Again, no BRE-gal reporter activity was detected in the allantois (Figure 8, panels A2, B2, C2, D2).

At early headfold stage, our data also showed that the neural ectoderm (ne) is free of reporter gene activity (Figure 8, panel B1). By late headfold stage, weak reporter gene expression was observed in the headfold mesenchyme (ms), but was still absent in the ne (Figure 8, panel D1). This was consistent with previous studies that demonstrated Bmp antagonism promoting neural fates, which was first shown in *Xenopus* [4], [71]; and in mES cells, Bmp4 inhibited neural differentiation [72].

Our BRE-gal indicator mice showed activity in the posterior primitive streak (ps, Figure 8, panels B3, D3), specifically in the eec at, and surrounding, the ps, and in the surface ectoderm and somatopleure (ses). Bmp4 transcripts were observed in the posterior ps, and analysis of Bmp4-deficient embryos implicated its role in proliferation and differentiation of embryonic and extraembryonic mesoderm [67]. Because the location of BRE-gal activity was exclusively in the ectoderm rather than the mesoderm, this raises the possibilities that Bmp activity within the mesoderm is not regulated by the BRE, or that Bmp signaling is absent in the mesoderm.

At late headfold stage, BRE-gal activity was not detected in the cardiac crescent (Figure 8, panels C1, C2, D1). However there is ample evidence showing that Bmp signaling is involved throughout heart development [35], [36], [37]. The lack of BRE-gal activity at this time suggests that cardiac crescent cells either utilize BRE-independent transcriptional machinery, or the level of Bmp signaling is low such that it cannot be detected by our BRE-gal indicator. However, after embryonic turning has completed, we detected BRE-gal activity in heart structures at E8.75 (Figure 5, panel B1), implicating differential Bmp-dependent transcriptional regulation during heart morphogenesis, or the presence of higher levels of Bmp signaling activity.

### Bmp signaling versus BRE reporter

Based on extensive BRE-gal expression analysis, it is clear that our BRE-gal reporter does not capture all sites of Bmp signaling. This is likely caused by two reasons. First, due to moderate sensitivity of our BRE-gal based reporter in mES cells, the reporter may fail to capture low levels of Bmp signaling activity. Second, given the diverse modes of Smad interactions with gene promoters [11], [73], [74], it is unlikely that any single cis-regulatory sequence motif would satisfy the full array of Smad binding combinations with various transcription factors. Additional transcriptional co-factors could lend another level of complexity in how activated Smads regulate Bmp target genes. Furthermore, although Bmp ligands typically transduce their signal through Smads 1, 5, and 8, there is a non-canonical Bmp pathway(s) that is independent of the Smad proteins [75], [76], and this cannot be detected by our BRE-gal reporter. Despite these limitations, our BRE-gal mice are able to indicate many dynamic spatiotemporal activity changes associated with Bmp signaling during pre-primitive streak through mid-gestation stage development in the mouse.

Throughout this paper, we highlight various embryonic time points (E5.5–E13.5) and structures to discuss the BRE-gal expression patterns (Figures 3–8) and compare them with published studies regarding Bmp signaling. In many cases, like the pharyngeal arches and AER, our findings are in accord with other research. However in some cases, we observed no reporter gene expression, such as in the primordial germ cells at e6.5 (Figure 7) and in the cardiac crescent at headfold stages (Figure 8). A likely explanation is that formation of these tissues does not rely on BRE-mediated Bmp activity and/or requires lower levels of Bmp signaling activity that cannot be registered by the current BRE-gal reporter. We feel that these are not a shortcoming of the BRE-gal tool, but rather it reveals the intricacies of how highly-conserved signal transduction pathways are regulated.

### Co-opting of BRE-mediated Bmp signaling in extraembryonic tissues

In mouse embryos prior to and during the primitive streak stages, our BRE-gal reporter showed Bmp activity in the proximal extraembryonic visceral endoderm (Figure 7). Previous studies regarding Bmp signaling in the extraembryonic tissues of the early gastrula typically concentrated on how Bmp ligands influenced axis patterning, germ layer specification, and primordial germ cell induction in the embryo. However, BRE-gal activity was detected in the extraembryonic visceral endoderm at E5.5–E6.5 (Figure 7), and was still in extraembryonic tissues at E7.5–E8.0 (Figure 8). Our reporter therefore suggests that BRE-mediated Bmp activity is important within these extraembryonic tissues. Because extraembryonic visceral endoderm contributes to the placenta, it is tempting to speculate that our BRE-gal model reports a tissue-specific role for Bmp signaling in placental formation. In further support of our model, Smad1 transcripts were detected in the extraembryonic visceral endoderm at e6.5 [63]. Moreover, Smad1-deficient mouse embryos died at approximately E10.5 due to failure in placental connection [63].

Extraembryonic BRE-gal reporter activity continued into the headfold stages (E7.5–E8.0), although it was no longer observed in the extraembryonic visceral endoderm. Rather, reporter gene expression was seen in the chorionic dome, amniotic fold, and amnion (Figure 8). In agreement with our findings, *Bmp2* expression was observed in the amnion and chorion [67], [68], and Bmp2-null embryos died between E7.5–E9.0, which is the period when the placenta normally formed. This lethality was due to a defective amnion and chorion, along with disrupted heart formation [68]. Bmp2 thus holds an important role in proper cardiac development of the embryo and formation of the extraembryonic structures.

### Comparison of different BRE reporters

While our BRE sequence is derived from the *Xenopus id3* gene, another group previously identified a similar but different Bmp response element in the mouse *Id1* promoter [77] (Figure S3). Their studies indicated two short sequences (−1105/−1080 and −1052/−1032) that are most important for Bmp response, and fusion of these sequences comprises their defined Bmp-response element (which they also termed “BRE”). For their luciferase and ß-galactosidase reporter assays, two BRE sequences were fused (in reverse orientation to each other) and placed upstream of the minimal adenovirus major late promoter [77], [78]. This group generated BRE reporter mES cells (BRE-*luc*, BRE-*lac1*, and BRE-*lac2*), and BRE reporter mice (BRE-*lac1* and BRE-*lac2*). Their ES cell reporters responded to various Bmp ligands in a dose-dependent manner. Upon careful comparison between their BRE and our BRE reporter lines both in zebrafish and mouse, we note several differences in expression patterns. First, our transgenic embryos seem to reveal more sites of BRE-regulated Bmp activity, such as the chorion (Figure 8), otic vesicle, and somites (Figure 3, panel B1). Second, our BRE-gal ES cells respond quite uniformly to concentrations as low as 5 ng/ml of Bmp4 (Figure 2C, data not shown), compared to the non-uniform reporter response of BRE-*lac1* mES cells to 20 ng/ml of Bmp4 [78], suggesting that our BRE-gal lines are more sensitive and uniform in response toward Bmp treatment. While the difference in ligand concentrations could be due to the potency of commercially available Bmp ligands, the increased sensitivity of our construct is likely due to the presence of seven copies of our BRE in the BRE-gal reporter, rather than two copies used by the other group (BRE-*luc,* BRE-*lac1*, and BRE-*lac2*). In support of this notion, when we examined the threshold response to our BRE-gal reporter using different copy numbers of BRE, we found that the threshold sensitivity and amplitude response improved significantly with increased BRE copy numbers (unpublished data and [10]). This raises an interesting possibility to develop a series of Bmp reporters that may respond to different thresholds of Bmp ligand concentrations, a useful reagent for monitoring Bmp gradient activity in living embryos.

## Conclusions

Our BRE-gal mice show dynamically regulated expression patterns of Bmp signaling during mouse development. The fact that BRE-gal expression is detected in a multitude of cell types throughout various embryonic stages raises an interesting question about the role of our BRE. Can the BRE be shared among several different target genes to coordinate their expression? We currently do not have the answer to this question, but a few key points suggest the BRE may be an important cis-regulatory module used to coordinate expression of various target genes upon Bmp stimulation: First, our BRE is present in the promoter regions of *Id* and *Ventx* family genes, and *Flrt3*, and *Bmp2*. Second, the BRE is conserved from flies to humans. Third, in mES cells, many direct gene targets of Bmp4 share BRE sequences in their regulatory regions (data not shown). Our future analysis will involve identifying other Bmp-responsive elements to further uncover the intricacy of Bmp signaling. Lastly, while we analyzed various tissues and organs throughout different stages of mouse embryogenesis, we could not include all sites of BRE-gal reporter activity such as the liver, kidneys, bones, pancreas, and skin. Further studies will likely uncover additional roles for BRE-mediated regulation in development and maintenance of tissues.

## Materials and Methods

### Ethics Statement

All animal studies were approved and carried out according to the Institutional Animal Care and Use Committee (IACUC) guidelines at the University of California, Irvine under the protocol number 2008-2814. Mice were euthanized prior to surgery, and all efforts were made to minimize suffering.

### Generation of BRE-gal reporter construct and transgenic mES cell lines

The *7xBRE-LacZ* reporter gene was created by digesting *7xBRE Xid3-pCX GFP3* [10] with *Hin*dIII and *Spe*I. Once the *7xBRE* concatamer and *Xid3* (−201/+70) minimal promoter were isolated, this fragment was subcloned between the HindIII and XbaI sites of the pBAT-Gal construct, thereby replacing the *siamois* minimal promoter and Lef/Tcf sites [21]. The resulting construct is *7xBRE-201Xid3-nlsLacZ/PGKNeo*, also referred to as BRE-gal. Reporter mES cell lines were generated by electroporation of the linearized BRE-gal DNA fragment into feeder-independent E14 mES cells derived from a 129P2/Ola background [79]. Neomycin-resistant colonies were subjected to Southern blot analysis to verify single-copy integration. Karyotype analysis was then performed on six colonies to confirm normal chromosome count, and two BRE-gal mES cell lines were established.

### Maintenance of BRE-gal mES cells

BRE-gal E14 mES cell lines were cultured on 0.1% gelatin pre-coated tissue culture dishes in Glasgow Minimum Essential medium (Sigma), containing LIF, 10% FBS, non-essential amino acids, 100 mM sodium pyruvate, 200 mM glutamine, 2-mercaptoethanol. Cells were grown at 37°C in 5% CO_2_, and split every other day using a solution containing 0.5% Trypsin-EDTA (Invitrogen) and chicken serum (Invitrogen) in PBS.

### X-gal staining and ONPG assays of BRE-gal mES cells

BRE-gal mES cells were treated with Bmp2, 4 and 7 homodimers, and Bmp4/7 heterodimer (R&D) for 24 hours at 37°C in 5% CO_2_. After treatment, cells were fixed (5 mM EGTA, 2 mM magnesium chloride, 0.05% glutaraldehyde in PBS) for 15 minutes, washed with PBS, and incubated in a staining buffer containing 1 mg/ml of 5-bromo-4-chloro-3-indolyl-β-d-galactopyranoside (X-gal) for 4 hours at 37°C.

BRE-gal mES cells were treated with Bmp2 and 4 at 5 ng/ml and 20 ng/ml, and Fgf4, Wnt3A, Gdf3, Activin, Tgfβ1 at 20 ng/ml for 24 hours at 37°C. Cells were lysed, and lysates were mixed with a reaction solution containing 4 mg/ml of ortho-nitrophenyl-b-galactopyranoside (ONPG). The lysate mixtures were incubated at 37°C for 15–20 minutes, and the reactions were stopped with 50 ml of 1 M Na_2_CO_3_. The optical density (OD) of the colorimetric reaction was measured with a spectrophotometer at 405 nm.

### Luciferase Assays

The BRE(7X)-*luc* wild type (WT) and BRE(7X)-*luc* mutant (MT) constructs were each co-transfected with the *pCMV-β-galactosidase* plasmid into wildtype E14 mES cells using Lipofectamine 2000 (Invitrogen). As a positive control, the *pCMV-luc* construct was also transfected into wildtype E14 mES cells. Three biological replicates for each DNA transfection were prepared. Cell media was changed 6–8 hrs post-transfection, then cells were harvested 24 hours post-transfection and subjected to a luciferase assay.

### Establishment of transgenic mouse lines

Reporter mice were generated by two different methods: (1) pronuclear injection of the BRE-gal DNA construct, and (2) blastocyst injection of BRE-gal mES cells. In the first method, a linearized BRE-gal DNA construct was injected into pronuclei of fertilized mouse eggs. Genotyping for the *lacZ* gene identified nine transgenic mice, which were then crossed to CD1 mice to generate F1 progeny. Genotyping for *lacZ* in the F1 progeny confirmed germline transmission and two founder mice were used to establish BRE-gal reporter lines. Embryos were incubated in a staining solution containing 1 mg/ml of X-gal. Analysis at various embryonic stages confirmed similar X-gal staining patterns between the two lines in the pharyngeal arches, eyes, and forebrain/telencephalon (compare Figure 3, panels B1–B2, Figure S4, and data not shown). In the second method, BRE-gal mES cells were injected into mouse blastocysts for implantation into foster mothers. Chimeras were mated to CD1 mice to test for germline transmission of the reporter gene. Two chimeras founded BRE-gal reporter mouse lines, and embryonic analysis at various stages confirmed that the X-gal staining pattern is consistent between the two lines for several generations. Importantly, overall X-gal staining patterns observed between mice generated from pronuclear injection and from blastocyst injection of BRE-gal mES cells were very similar.

### Wholemount X-gal staining and histology of BRE-gal mouse embryos

For the analysis presented in this manuscript, BRE-gal male mice were mated with CD1 females, and embryos of various stages (E5.5–E13.5) were collected from pregnant CD1 females. Dissections were performed in ice-cold 1× PBS. Embryos were fixed in ice-cold 4% PFA, and subjected to wholemount X-gal staining for 1–2 hours or overnight at 37°C. BRE-gal males with one copy of the transgene produced litters with transgenic and non-transgenic embryos in Mendelian ratios. Non-transgenic embryos consistently any lacked staining (data not shown). This internal control validated that the staining pattern observed in transgenic embryos was indeed due to ß-galactosidase expressed from the integrated reporter.

For sectioning on a microtome, X-gal-stained embryos were incubated in Bouin's Fixative (75% saturated picric acid, 20% formaldehyde, 5% glacial acetic acid) at room temperature, gently shaking for 16–18 hours. After washing in 1× PBS, embryos were serially dehydrated to 100% ethanol. Embryos were washed in toluene before embedding in paraplast for sectioning on a microtome at 10 µm thickness. Paraffin sections were mounted on SuperFrost slides (Fisher Scientific) and air dried overnight. The slides were again serially dehydrated to 100% ethanol, then de-paraffinized with Histoclear (National Diagnostics). The slides were sealed with coverslips using Permount (Fisher Scientific). For sectioning on a cryotome, X-gal-stained embryos were cryoprotected in a stepwise fashion to 30% sucrose in 1× PBS at 4°C. Embryos were embedded in OCT Compound (Tissue Tek) for sectioning at 10 µm thickness. Cryosections were mounted on SuperFrost slides and incubated at 45–50°C for 30–60 min. Cryosections were serially dehydrated to 100% ethanol, then incubated in Histoclear for a few minutes. The slides were sealed with coverslips using Permount.

## Supporting Information

Figure S1 BRE-mediated responsiveness is evolutionary conserved between zebrafish, frog, and mouse. Three transgenic BRE reporter embryos are shown to demonstrate the similar responsiveness to BRE-mediated Bmp signaling activity, particularly in the brain, eyes, heart, and pharyngeal arches, and somites. A wholemount X-gal stained BRE-gal mouse embryo at E9.5 is shown in a left, lateral view with anterior at the top (A). A BRE-gfp *Xenopus laevis* embryo that has undergone *in situ* hybridization for GFP transcripts is shown in a left, lateral view with anterior to the left (B). A BRE-gfp zebrafish embryo is shown in a left, lateral view with anterior to the left (C). Abbreviations: e, eye; h, heart; hb, hindbrain; pa, pharyngeal arches; pah, region of pharyngeal arches and heart; s, somites.
(TIF)

Figure S2 BRE-gal reporter activity in the forelimb bud of a mouse embryo at E11.0. The AER shown in (A) divides the ventral and dorsal portions of the limb bud, with ventral on the left and dorsal on the right. A dorsal view of the forelimb is shown in (B), with a corresponding transverse section that is slightly oblique (C). In both (B) and (C), the AER is on the left, and anterior is at the top. The asterisk indicates the darker, posterior stripe of X-gal staining in the limb mesenchyme. Section thickness is 12 µm. Abbreviations: aer, apical ectodermal ridge; ms, mesenchyme.
(TIF)

Figure S3 Comparison of the BRE sequences from the *Xenopus id3* and mouse *Id1* promoter regions. Two, short Bmp-responsive sequences from the mouse *Id1* regulatory region (−1105/−1080 and −1052/−1032) were designated as the BRE by Korchynskyi and ten Dijke [77]. Comparison of the (−1052/−1032) mouse *Id1* BRE fragment shows that the sequence is similar, but different from the *Xid3* BRE previously characterized by us [76]. In the *Xid3* BRE, the Smad1 binding site and SBE are underlined.
(TIF)

Figure S4 A BRE-gal mouse line generated by pronuclear injection. An independent mouse line was also established by pronuclear injection of the BRE-gal DNA construct. A BRE-gal embryo at E9.5 is shown to demonstrate the similarity in overall X-gal staining patterns in the pharyngeal arches, eyes, and forebrain. It should be noted that staining in the forebrain, midbrain, and hindbrain is present, however it is weaker than staining in embryos from blastocyst injection. The head is shown in a left, lateral view (A), an oblique, front view (B), and a dorsal view (C).
(TIF)
