# Supplementary figures and images for: Bmp Indicator Mice Reveal Dynamic Regulation of Transcriptional Response
Source: PLoS One. 2012 Sep 11;7(9):e42566. doi: 10.1371/journal.pone.0042566 (PMC3439458; doi:10.1371/journal.pone.0042566)

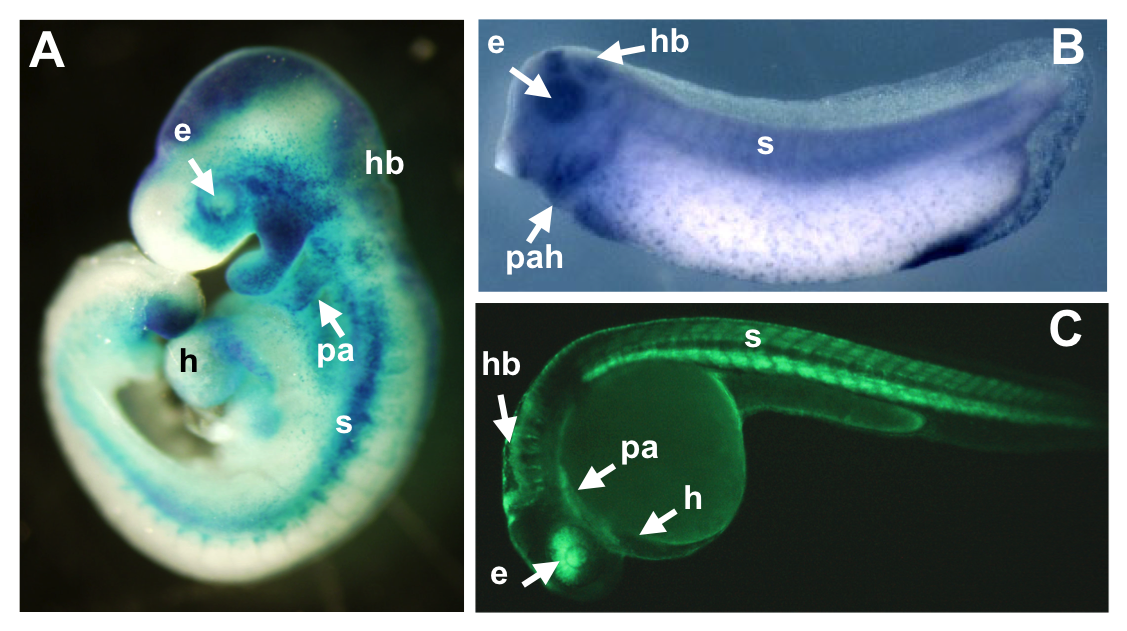

Supplement: Figure S1 — BRE-mediated responsiveness is evolutionary conserved between zebrafish, frog, and mouse. Three transgenic BRE reporter embryos are shown to demonstrate the similar responsiveness to BRE-mediated Bmp signaling activity, particularly in the brain, eyes, heart, and pharyngeal arches, and somites. A wholemount X-gal stained BRE-gal mouse embryo at E9.5 is shown in a left, lateral view with anterior at the top (A). A BRE-gfp Xenopus laevis embryo that has undergone in situ hybridization for GFP transcripts is shown in a left, lateral view with anterior to the left (B). A BRE-gfp zebrafish embryo is shown in a left, lateral view with anterior to the left (C). Abbreviations: e, eye; h, heart; hb, hindbrain; pa, pharyngeal arches; pah, region of pharyngeal arches and heart; s, somites. (TIF) [file pone.0042566.s001.tif]

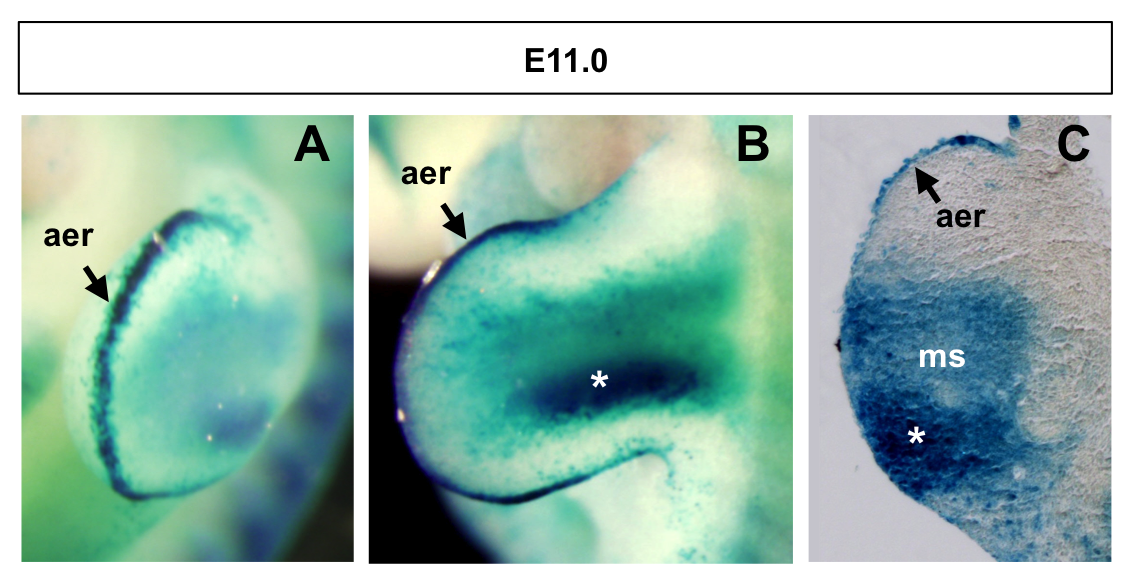

Supplement: Figure S2 — BRE-gal reporter activity in the forelimb bud of a mouse embryo at E11.0. The AER shown in (A) divides the ventral and dorsal portions of the limb bud, with ventral on the left and dorsal on the right. A dorsal view of the forelimb is shown in (B), with a corresponding transverse section that is slightly oblique (C). In both (B) and (C), the AER is on the left, and anterior is at the top. The asterisk indicates the darker, posterior stripe of X-gal staining in the limb mesenchyme. Section thickness is 12 µm. Abbreviations: aer, apical ectodermal ridge; ms, mesenchyme. (TIF) [file pone.0042566.s002.tif]

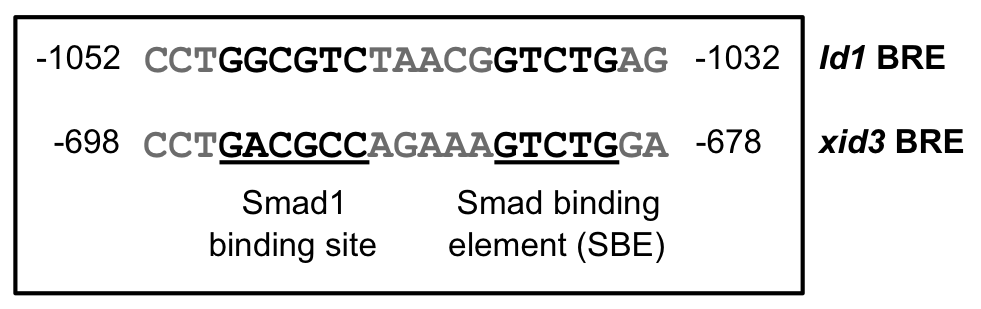

Supplement: Figure S3 — Comparison of the BRE sequences from the Xenopus id3 and mouse Id1 promoter regions. Two, short Bmp-responsive sequences from the mouse Id1 regulatory region (−1105/−1080 and −1052/−1032) were designated as the BRE by Korchynskyi and ten Dijke [77]. Comparison of the (−1052/−1032) mouse Id1 BRE fragment shows that the sequence is similar, but different from the Xid3 BRE previously characterized by us [76]. In the Xid3 BRE, the Smad1 binding site and SBE are underlined. (TIF) [file pone.0042566.s003.tif]

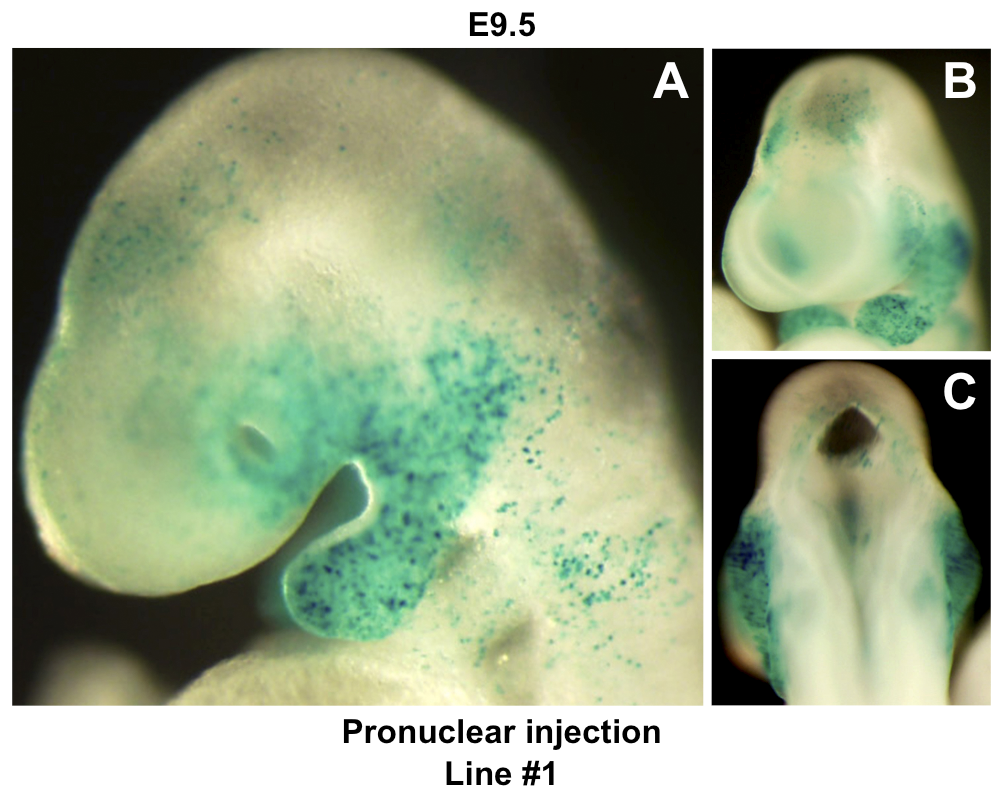

Supplement: Figure S4 — A BRE-gal mouse line generated by pronuclear injection. An independent mouse line was also established by pronuclear injection of the BRE-gal DNA construct. A BRE-gal embryo at E9.5 is shown to demonstrate the similarity in overall X-gal staining patterns in the pharyngeal arches, eyes, and forebrain. It should be noted that staining in the forebrain, midbrain, and hindbrain is present, however it is weaker than staining in embryos from blastocyst injection. The head is shown in a left, lateral view (A), an oblique, front view (B), and a dorsal view (C). (TIF) [file pone.0042566.s004.tif]
